# Supplementary material for: The effects of visual stimulation on the cortical activity of brainstem stroke dysphagia patients: A functional near-infrared spectroscopy study
Source: PLoS One. 2025 Jun 6;20(6):e0325510. doi: 10.1371/journal.pone.0325510 (PMC12143532; doi:10.1371/journal.pone.0325510)
Supplement: S1 Table — (DOCX) [file pone.0325510.s001.docx]

| **TABLE 1. Baseline demographic and clinical characteristics.** | | | | |
| --- | --- | --- | --- | --- |
| Characteristic | HC group（N=16） | Patient group（N=30） | *T/Z-*value | *P-*value |
| Age  (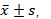years) | 58.9±5.9 | 61.9±11.9 | 1.1387^a^ | 0.173 |
| MMSE  [*M*(*P*_25_*,P*_75_)] | 30.0（29.0,30.0） | 28.0（26.3，30.0） | -2.684^b^ | 0.007* |
| VAS  (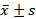) | 8.70±1.1 | 8.58±1.8 | 0.242 | 0.81 |
| SNAQ  (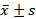) | 16.5±1.4 | 16.0±2.8 | 0.702 | 0.486 |
