# Supplementary material for: The effects of visual stimulation on the cortical activity of brainstem stroke dysphagia patients: A functional near-infrared spectroscopy study
Source: PLoS One. 2025 Jun 6;20(6):e0325510. doi: 10.1371/journal.pone.0325510 (PMC12143532; doi:10.1371/journal.pone.0325510)
Supplement: S2 Table — (DOCX) [file pone.0325510.s002.docx]

| **TABLE 2. Intergroup comparison of brain area activation in food visual stimulation tasks.** | | | | |
| --- | --- | --- | --- | --- |
| Channel | Label of SD | Brain region | *T*-value | *P*-value |
| 1 | S1-D1 | Middle Temporal gyrus-R | 0.641 | 0.681 |
| 2 | S1-D6 | Superior Temporal Gyrus-R | 0.436 | 0.798 |
| 3 | S2-D1 | Temporopolar area-R | 1.331 | 0.315 |
| 4 | S2-D2 | Inferior prefrontal gyrus-R | 2.194 | 0.124 |
| 5 | S2-D7 | Inferior prefrontal gyrus-R | 2.037 | 0.136 |
| 6 | S3-D2 | Frontopolar area-R | 2.355 | 0.097 |
| 7 | S3-D3 | Orbitofrontal area-R | 2.575 | 0.081 |
| 8 | S3-D8 | Frontopolar area-N | 3.092 | 0.043^*^ |
| 9 | S4-D3 | Orbitofrontal area-L | 2.658 | 0.075 |
| 10 | S4-D4 | Orbitofrontal area-L | 3.109 | 0.043^*^ |
| 11 | S4-D9 | Frontopolar area-L | 2.803 | 0.056 |
| 12 | S5-D4 | Temporopolar area-L | 1.696 | 0.222 |
| 13 | S5-D5 | Middle Temporal gyrus-L | -0.263 | 0.866 |
| 14 | S5-D10 | Superior Temporal Gyrus-L | 0.469 | 0.798 |
| 15 | S6-D5 | Middle Temporal gyrus-L | -0.391 | 0.798 |
| 16 | S6-D11 | Middle Temporal gyrus-L | 0.047 | 0.963 |
| 17 | S7-D1 | Superior Temporal Gyrus-R | 1.037 | 0.473 |
| 18 | S7-D6 | Subcentral area-R | 0.120 | 0.925 |
| 19 | S7-D7 | Broca's area-R | 1.498 | 0.261 |
| 20 | S7-D12 | Pre-Motor and Supplementary Motor Cortex-R | 1.540 | 0.261 |
| 21 | S8-D2 | Frontopolar area-R | 0.702 | 0.648 |
| 22 | S8-D7 | Dorsolateral prefrontal cortex-R | 0.393 | 0.798 |
| 23 | S8-D8 | Frontopolar area-R | 1.334 | 0.315 |
| 24 | S8-D13 | Dorsolateral prefrontal cortex-R | -0.166 | 0.906 |
| 25 | S9-D3 | Frontopolar area-N | 1.999 | 0.136 |
| 26 | S9-D8 | Frontopolar area-R | 2.344 | 0.097 |
| 27 | S9-D9 | Frontopolar area-L | 3.075 | 0.043^*^ |
| 28 | S9-D14 | Frontopolar area-N | 2.388 | 0.097 |
| 29 | S10-D4 | Inferior prefrontal gyrus-L | 1.372 | 0.315 |
| 30 | S10-D9 | Frontopolar area-L | 1.759 | 0.205 |
| 31 | S10-D10 | Broca's area-L | 0.459 | 0.798 |
| 32 | S10-D15 | Dorsolateral prefrontal cortex-L | 2.334 | 0.097 |
| 33 | S11-D5 | Middle Temporal gyrus-L | -0.225 | 0.878 |
| 34 | S11-D10 | Superior Temporal Gyrus-L | 0.291 | 0.863 |
| 35 | S11-D11 | Primary and Auditory Association Cortex-L | 0.737 | 0.648 |
| 36 | S11-D16 | Subcentral area-L | 2.105 | 0.136 |
| 37 | S12-D7 | Broca's area-R | 0.719 | 0.648 |
| 38 | S12-D12 | Dorsolateral prefrontal cortex-R | 0.964 | 0.511 |
| 39 | S12-D13 | Dorsolateral prefrontal cortex-R | 2.891 | 0.057 |
| 40 | S13-D8 | Frontopolar area-R | 1.498 | 0.261 |
| 41 | S13-D13 | Dorsolateral prefrontal cortex-R | 1.599 | 0.244 |
| 42 | S13-D14 | Dorsolateral prefrontal cortex-R | 1.286 | 0.328 |
| 43 | S14-D9 | Frontopolar area-L | 2.023 | 0.136 |
| 44 | S14-D14 | Dorsolateral prefrontal cortex-L | 2.020 | 0.136 |
| 45 | S14-D15 | Dorsolateral prefrontal cortex-L | 3.431 | 0.043^*^ |
| 46 | S15-D10 | Broca's area-L | 1.669 | 0.223 |
| 47 | S15-D15 | Dorsolateral prefrontal cortex-L | 1.980 | 0.136 |
| 48 | S15-D16 | Pre-Motor and Supplementary Motor Cortex-L | 0.712 | 0.648 |
