# Supplementary material for: The effects of visual stimulation on the cortical activity of brainstem stroke dysphagia patients: A functional near-infrared spectroscopy study
Source: PLoS One. 2025 Jun 6;20(6):e0325510. doi: 10.1371/journal.pone.0325510 (PMC12143532; doi:10.1371/journal.pone.0325510)
Supplement: S3 Table — (DOCX) [file pone.0325510.s003.docx]

| **TABLE 3.** **Intergroup comparison of ROI functional connectivity in resting tasks.** | | |
| --- | --- | --- |
| ROI Brain region | T | P |
| Broca’s area-R- Frontopolar area-L | -2.767 | 0.046^*^ |
| Broca’s area-R - Frontopolar area-N | -3.376 | 0.014^*^ |
| Broca’s area-R - Frontopolar area-R | -3.820 | 0.006^**^ |
| Dorsolateral prefrontal cortex-R - Frontopolar area-N | -3.582 | 0.010^*^ |
| Dorsolateral prefrontal cortex-R - Frontopolar area-R | -3.126 | 0.024^*^ |
| Inferior prefrontal gyrus-R - Dorsolateral prefrontal cortex-R | -4.908 | 0.000^**^ |
| Inferior prefrontal gyrus-R - Dorsolateral prefrontal cortex-L | -2.970 | 0.032^*^ |
| Inferior prefrontal gyrus-R - Frontopolar area-L | -4.024 | 0.004^**^ |
| Inferior prefrontal gyrus-R - Frontopolar area-N | -4.769 | 0.000^**^ |
| Inferior prefrontal gyrus-R - Frontopolar area-R | -4.851 | 0.000^**^ |
| Inferior prefrontal gyrus-R - Orbitofrontal area-L | -4.793 | 0.000^**^ |
| Inferior prefrontal gyrus-R - Orbitofrontal area-R | -3.106 | 0.024^*^ |
| Pre-Motor and Supplementary Motor Cortex-R - Frontopolar area-N | -2.937 | 0.032^*^ |
| Pre-Motor and Supplementary Motor Cortex-R - Frontopolar area-N | -3.504 | 0.011^*^ |
